# Supplementary material for: Full-Length Transcriptome Sequencing Reveals the Impact of Cold Stress on Alternative Splicing in Quinoa
Source: Int J Mol Sci. 2022 May 20;23(10):5724. doi: 10.3390/ijms23105724 (PMC9144462; doi:10.3390/ijms23105724)
Supplement: Supplementary file 1 [file ijms-23-05724-s001.zip › Table S1.pdf]

Table S1. Summary of ONT native RNA read statistics

Sample ID	Read Number	Number of clean reads (except rRNA)	N50	Mean Length	Max Length	Mapped rate	Mean Qscore
Cq-5C-1	8,511,940	8,453,061	949	919	12,926	99.62%	Q10
Cq-5C-2	9,048,712	8,992,927	1,133	1,069	11,039	99.74%	Q10
Cq-5C-3	5,940,290	5,891,433	1,066	1,021	26,092	99.69%	Q10
Cq-5N-1	5,785,901	5,752,023	1,182	1,084	12,877	99.60%	Q11
Cq-5N-2	6,842,001	6,800,504	1,297	1,158	14,466	99.67%	Q11
Cq-5N-3	8,257,027	8,206,792	883	862	8,772	99.67%	Q11
Cq-64C-1	7,742,358	7,700,378	1,135	1,068	13,771	99.49%	Q11
Cq-64C-2	7,386,724	7,344,636	1,086	1,040	10,049	99.58%	Q11
Cq-64C-3	7,649,928	7,606,285	1,014	988	12,123	99.65%	Q11
Cq-64N-1	7,803,098	7,761,440	945	911	12,734	99.40%	Q11
Cq-64N-2	7,798,482	7,747,811	1,028	979	11,409	99.35%	Q11
Cq-64N-3	7,551,981	7,515,583	1,052	997	10,553	99.50%	Q11
