# Supplementary material for: Full-Length Transcriptome Sequencing Reveals the Impact of Cold Stress on Alternative Splicing in Quinoa
Source: Int J Mol Sci. 2022 May 20;23(10):5724. doi: 10.3390/ijms23105724 (PMC9144462; doi:10.3390/ijms23105724)
Supplement: Supplementary file 1 [file ijms-23-05724-s001.zip › Zheng et al., figures S1-S6.pdf]

Figure S1

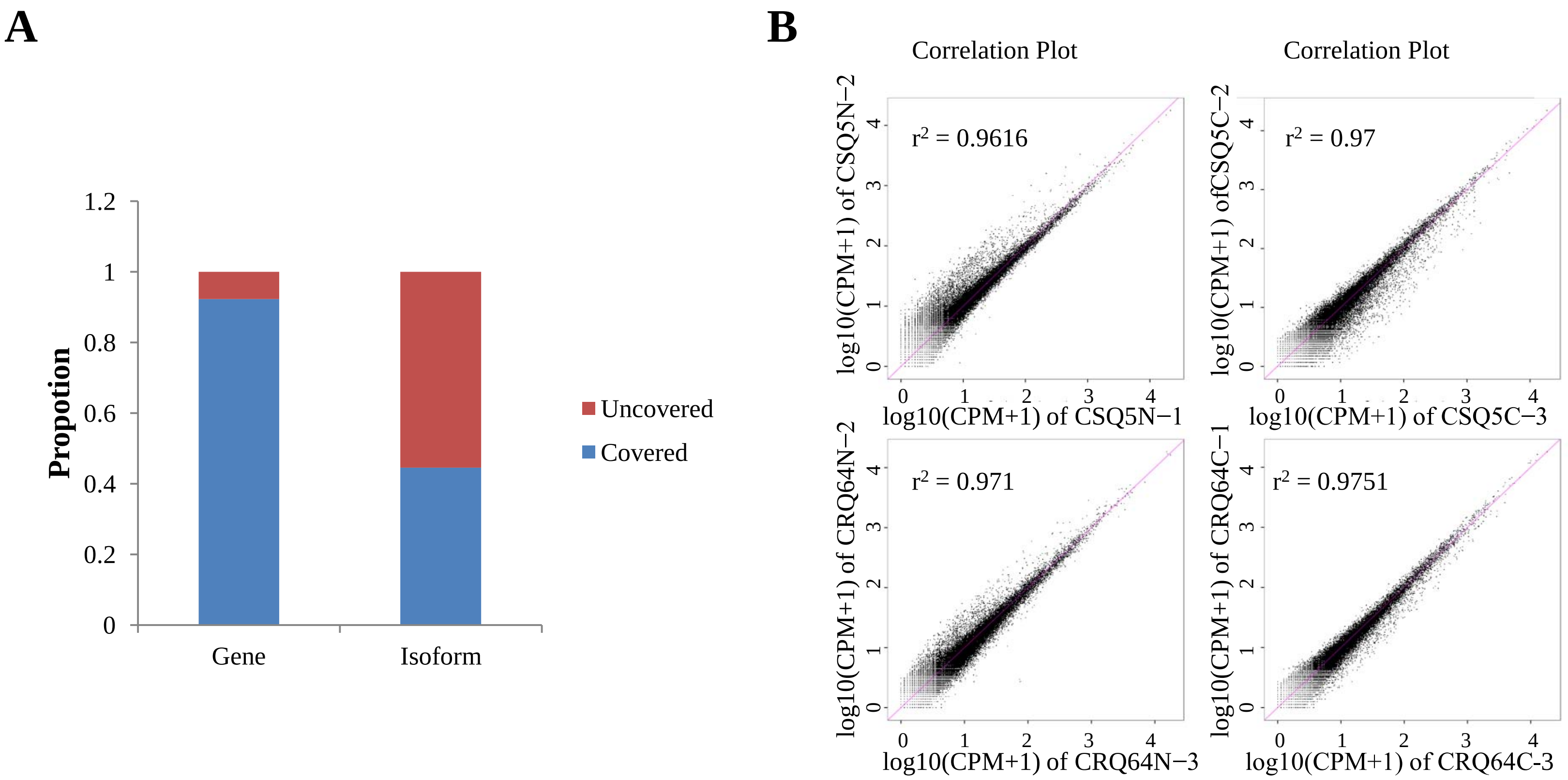

Figure S1: The proportion of the isoforms or genes covered on quinoa reference transcripts and correlation between two replicates of sequenced libraries. (a) The proportion of the isoforms or genes that covered reference transcripts; (b) correlation between two replicates of sequenced libraries. Shown are the biological replicates from CSQ5 (up) and CRQ64 (down).

Figure S2

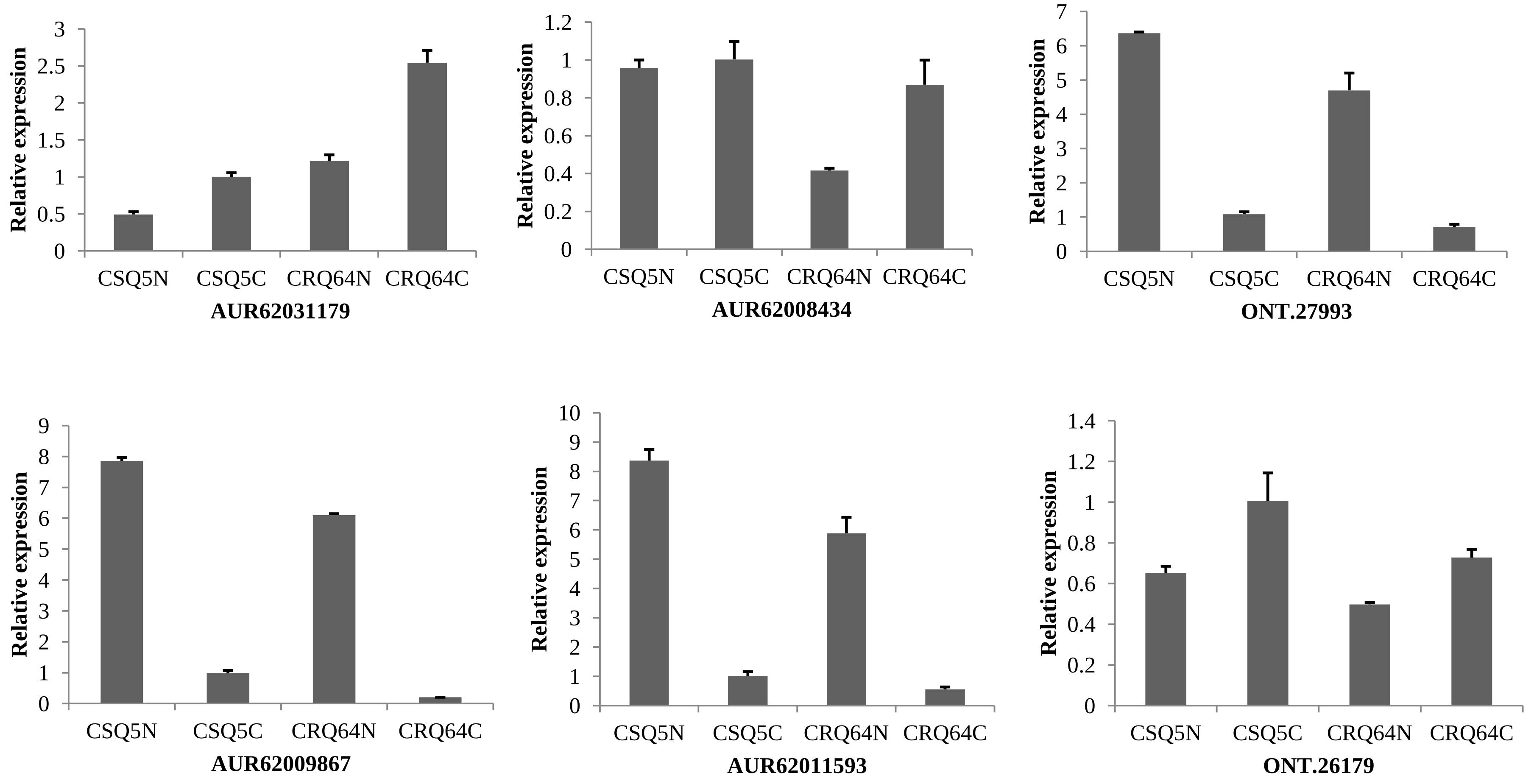

Figure S2: Validation of RNA-seq results by real-time RT-qPCR. The relative expression levels were calculated according to the 2-ΔΔCT method using actin as internal reference gene.

Figure S3

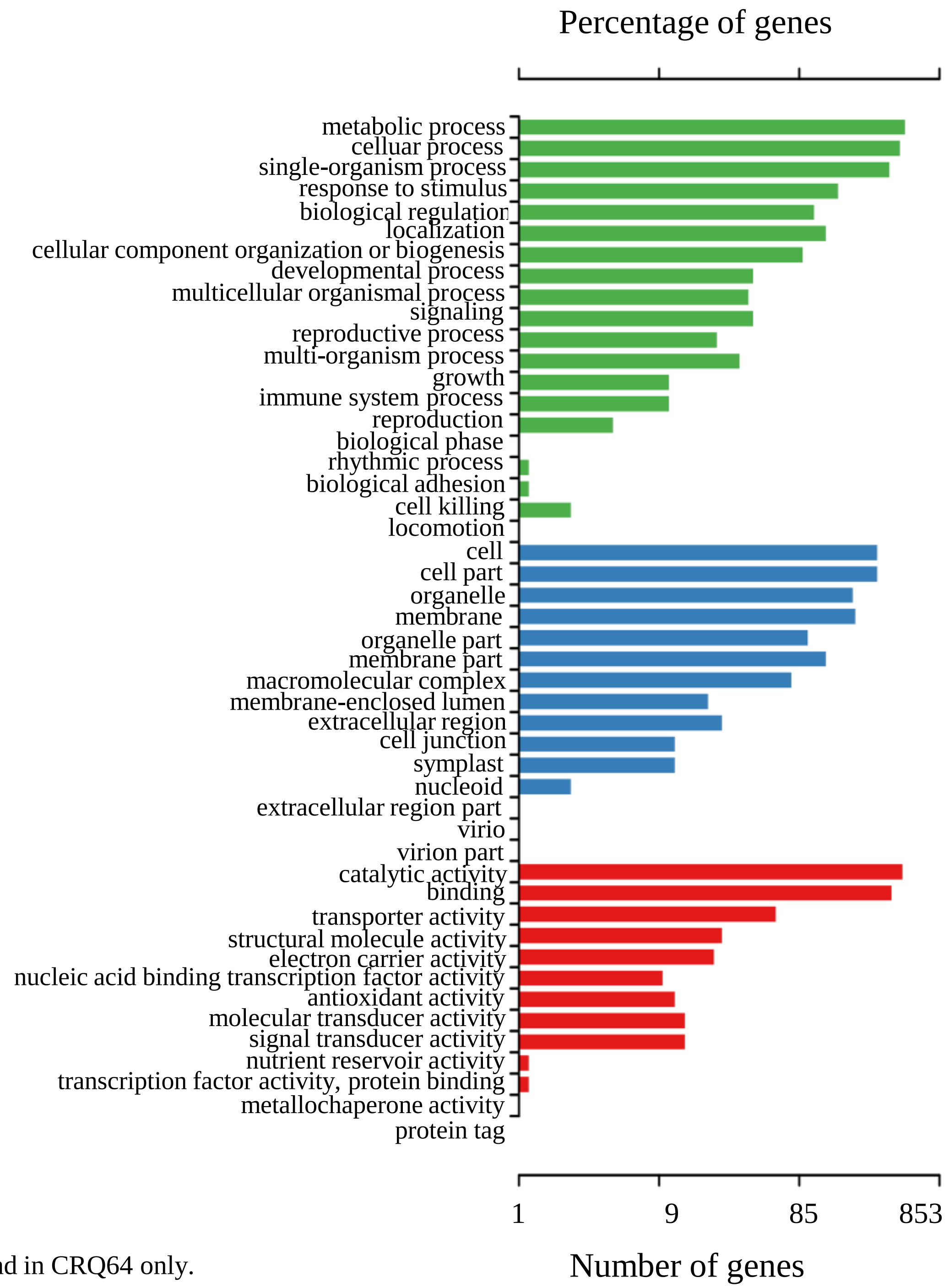

Figure S3. GO enrichment of DEGs found in CRQ64 only.

Figure S4

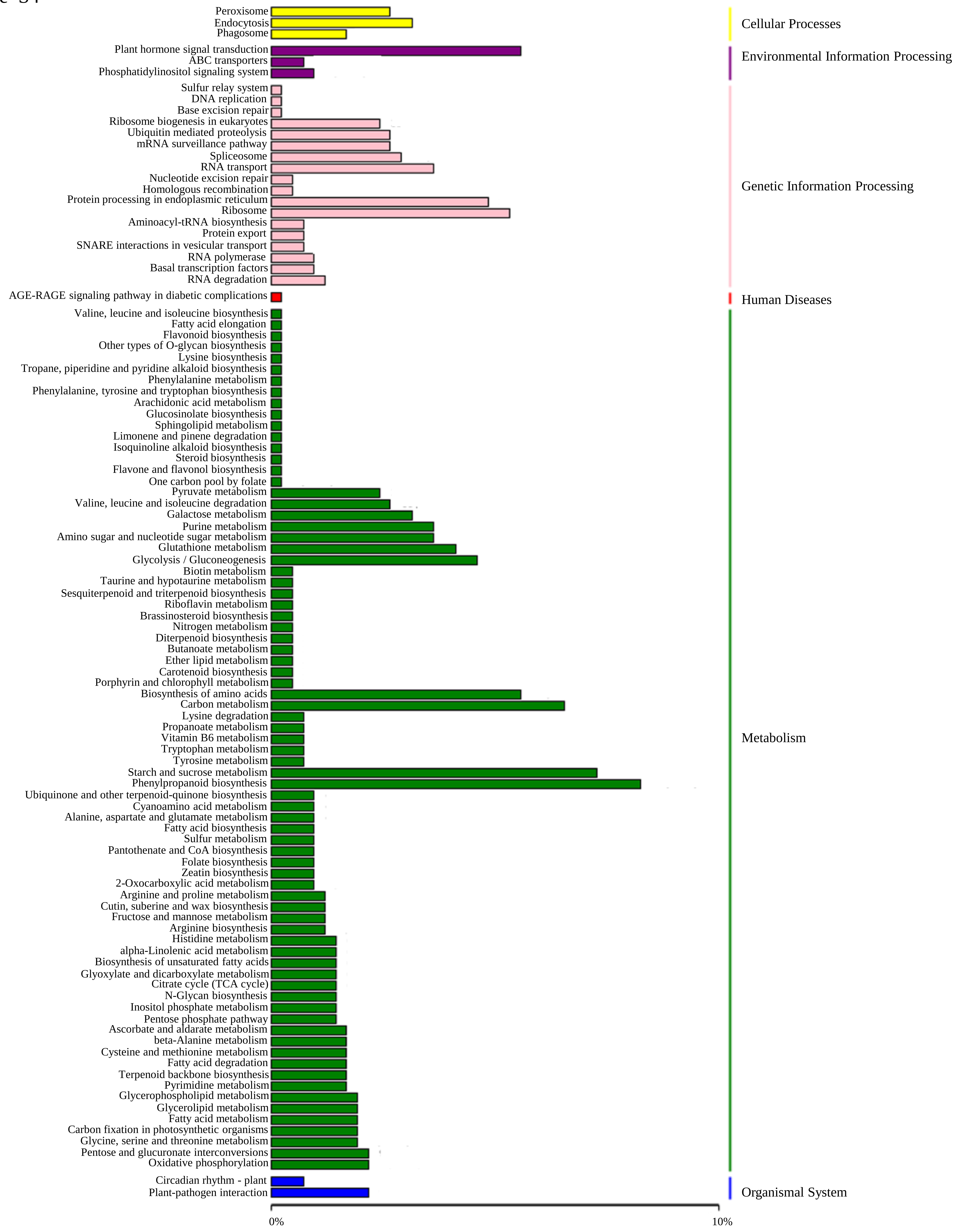

Figure S4. KEGG class of 2393 CRQ64-specific DEGs.

Figure S5

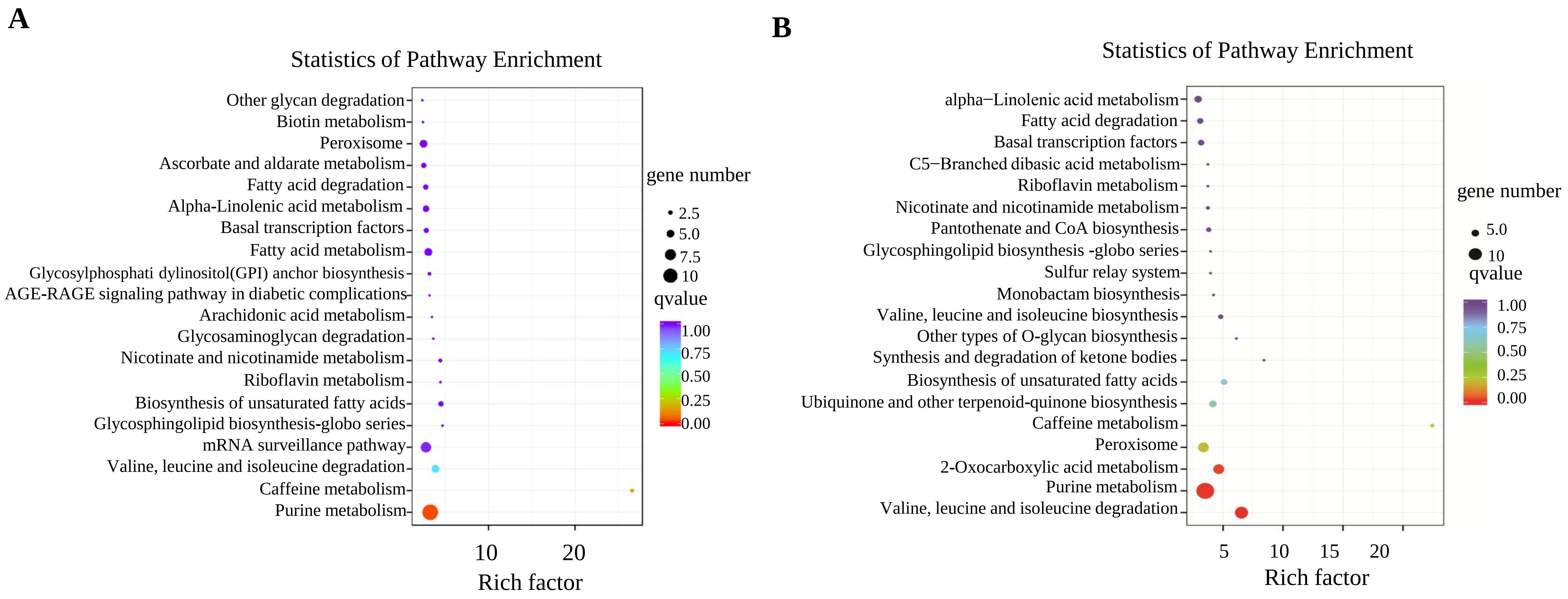

Figure S5. KEGG enrichment of the genes with DAS events in CSQ5 and CRQ64. (a) KEGG enrichment of the genes with DAS events between CSQ5N and CSQ5C; (b) KEGG enrichment of the genes with DAS between CSQ64N and CSQ64C.

Figure S6

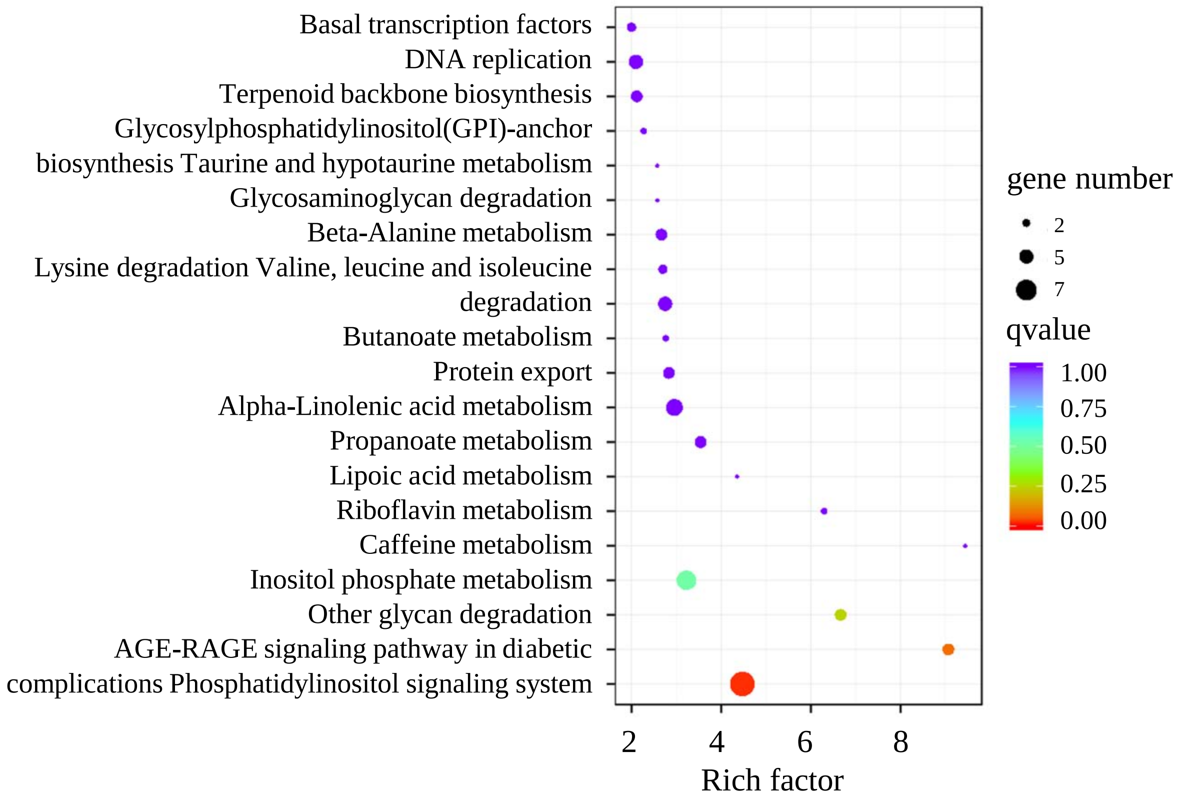

Figure S6. KEGG enrichment of target genes of 136 LncRNAs.
